# Editorial: Gynecological and gastrointestinal cancers: Recent advances in molecular diagnosis and targeted therapy

**DOI:** 10.3389/fmolb.2023.1167337

**Published:** 2023-03-03

**Authors:** Lan Zhang, Guan Wang, Lingjuan Zhu, Wei Wei, Jin Zhang

**Affiliations:** ^1^ Sichuan Engineering Research Center for Biomimetic Synthesis of Natural Drugs, School of Life Science and Engineering, Southwest Jiaotong University, Chengdu, China; ^2^ State Key Laboratory of Biotherapy and Cancer Center, Nursing Key Laboratory of Sichuan Province, Innovation Center of Nursing Research, West China Hospital, and Collaborative Innovation Center of Biotherapy, Sichuan University, Chengdu, China; ^3^ Key Laboratory of Structure-Based Drug Design and Discovery of Ministry of Education, School of Traditional Chinese Materia Medica, Shenyang Pharmaceutical University, Shenyang, China; ^4^ Blood Cell Development and Function Program, Fox Chase Cancer Center, Philadelphia, PA, United States; ^5^ School of Pharmaceutical Sciences, Health Science Center, Shenzhen University, Shenzhen, China

**Keywords:** breast cancer, pancreatic adenocarcinoma, colorectal cancer, prognosis, molecular mechanisms, targeted therapy

Cancer is often rendered an uncurable disease, which poses a great threat to human health worldwide. Among all kinds of cancers, gynecological cancers (including breast, ovarian, cervical, and endometrial cancer) and gastrointestinal cancers (including gastric, colorectal, liver, and pancreatic cancer) are the most common cancer types worldwide (Ciou et al., 2022; Murphy et al., 2018). Although chemotherapy, radiotherapy, and surgical excision are commonly used to treat these gynecological and gastrointestinal cancers, cancer cells frequently develop resistance to conventional cancer therapy due to a variety of mechanisms (Davern et al., 2020; Marchetti et al., 2021). Accordingly, new therapeutic strategies such as the identification of novel potential molecular targets or biomarkers, as well as the development of new molecular diagnostic approaches and targeted therapy (e.g., proteins, transcription factors, non-coding RNA, targeted small-molecule drugs, etc.) are greatly needed to overcome inherent and acquired resistance, which would provide a promising therapeutic approach for the treatment of various gynecological and gastrointestinal cancers.

Breast cancer is the most common global malignancy and one of the most serious gynecological cancers affecting women, which also represents currently a top biomedical research priority (Akram et al., 2017). And its incidence and mortality rates are expected to increase continuously the next years (Barzaman et al., 2020). Accumulating evidence suggests that circular RNAs (circRNAs) are highly correlated with tumor progression and pathogenesis in breast cancer (Sang et al., 2019). Whereas, their regulatory roles and corresponding mechanisms in breast cancer are still unknown to some extent. Therefore, Lin et al. explored the circRNA-mediated competive endogenous RNA (ceRNA) network to uncover the possible roles and clinical implications of circRNAs in breast cancer. Through functional experiments, they found that the depletion of two circRNAs, circ_0008812 and circ_0001583, could significantly inhibit the proliferation of MCF-7 cells, providing a new perspective into the molecular mechanisms of breast cancer pathogenesis.

Pancreatic adenocarcinoma (PAAD) is the most common primary malignancy of the pancreas and is a highly aggressive tumor that is almost uniformly lethal in humans (Ge et al., 2022). According to previous research, the APOBEC family is associated with development of a variety of cancers (Mertz et al., 2022). Duan et al. discovered that APOBEC1/3A/3G/3H showed significantly elevated expression in PAAD than para-cancerous or normal tissues, indicating they have the potential to serve as novel biomarkers or therapeutic targets. Furthermore, recent evidence uncovered by Duan et al. suggests that SUMO family member is closely related to the occurrence and development of PAAD, which can also be used as a new biomarker and therapeutic target for patients with PAAD.

Colorectal cancer (CRC) is the third most common cancer and the second leading cause of cancer-related deaths in the world (Dekker et al., 2019). In addition, the incidence of CRC is increasing continuously in some areas of the world (Issa et al., 2017). Yunos et al. identified specific druggable somatic mutations by using the whole-genome sequencing approach on Malaysian patients, which could be of great benefit especially to Malaysian CRC patients. To note, at least one druggable somatic alteration was identified in 88% of the Malaysian CRC patients. Among them were two frameshift mutations in RNF43 (G156fs and P192fs) predicted to have responsive effects against the Wnt pathway inhibitor. Collectively, this research highlighted the potential of an alternative treatment targeting Wnt/Beta Catenin signaling pathway using whole genome sequencing, providing a new idea for the treatment of colorectal cancer. In addition, high expression of long non-coding RNA small nucleolar RNA host genes (lncRNA SNHGs) was revealed to be positively associated with poor CRC prognosis by Luo et al. Thus, lncRNA SNHG could serve as a potential prognostic indicator and therapeutic target for CRC.

In summary, the Research Topic “*Gynecological and gastrointestinal cancers: Recent advances in molecular diagnosis and targeted therapy*” collects studies focused on finding novel biomarkers and therapeutic targets in various gynecological and gastrointestinal cancers. The articles in this Research Topic highlight important, interrelated Research Topic and will hopefully serve as a catalyst for further studies that pave the way for developing novel targeting therapeutic strategies for cancers and overcoming therapeutic resistance. Ultimately, we trust that these efforts may eventually help improve survival outcomes and prognoses for gynecological and gastrointestinal cancer patients in the future.
